# Effective Coverage of Rehabilitation for Adults with Chronic Primary Low Back Pain in Uganda

**DOI:** 10.3390/ijerph23060693

**Published:** 2026-05-23

**Authors:** Wouter De Groote, Yehu Taremwa, Antony Duttine, Dan Kajungu

**Affiliations:** 1 Rehabilitation Programme, Department for Noncommunicable Diseases and Mental Health, World Health Organization, 1211 Geneva, Switzerland; 2 Makerere University Centre for Health and Population Research & Iganga Mayuge HDSS, Makerere University, Iganga, Kampala P.O. Box 111, Uganda; taremwayehu@gmail.com (Y.T.); dan.kajungu@gmail.com (D.K.); 3 Department of Global Health, Stellenbosch University, Stellenbosch, Cape Town 7505, South Africa

**Keywords:** rehabilitation, coverage, universal health coverage, health services, quality, low back pain

## Abstract

**Highlights:**

**Public health relevance—How does this work relate to a public health issue?**

- Rehabilitation services are essential health services that should be made available to a population. Many people, however, have limited access to the required rehabilitation services, particularly in low- and middle-income countries (LMICs).
- Low back pain (LBP) accounts for 5% of all years lived with a disability in Uganda. As effective rehabilitation interventions are available to manage chronic primary low back pain, it would be meaningful to make them accessible through the Ugandan health system as part of universal health coverage (UHC).

**Public health significance—Why is this work of significance to public health?**

- For the first time globally, the WHO global tracer indicator for effective coverage of rehabilitation is measured at the population level using chronic primary low back pain as the tracer health condition.
- In Uganda, in 2023, a significant drop was observed from crude coverage (46%) to effective coverage (7.05%) of rehabilitation services for chronic primary low back pain.

**Public health implications—What are the key implications or messages for practitioners, policy-makers and/or researchers in public health?**

- Cross-sectional mixed-methods studies are needed to identify barriers to effective provision of rehabilitation services in rural Uganda.
- Understanding the extent to which health systems are delivering key interventions to those who will benefit from them and the factors that explain gaps in delivery are critical inputs to decision-making at the local, national and global levels. When reliable data become available from other countries, rehabilitation could be included in the WHO’s and other frameworks for measuring UHC.

**Abstract:**

In Uganda, in 2019, 6.8 million people experienced health conditions that are amenable to rehabilitation. This is largely due to musculoskeletal disorders such as low back pain (LBP). Measuring effective coverage of rehabilitation means assessing whether a population that needs rehabilitation services receives the interventions with sufficient quality to produce the desired health gain. This study reports on the first measurement of effective coverage of rehabilitation in Uganda and globally using chronic primary LBP as the tracer health condition. A population survey was conducted to administer the WHO global tracer indicator questions. The survey questions were used to identify respondents with chronic primary LBP experiencing limitations in functioning and to determine utilization of rehabilitation services. The WHO Disability Assessment Schedule (WHODAS) 2.0 12-item was used for the measurement of an improvement in functioning that is meaningful to service users. The questions were nested in the Iganga–Mayuge Health and Demographic Surveillance Site (IMHDSS) update round 22 in seven sub-counties in rural Eastern Uganda between June and September 2023. Data collection followed a training session on survey administration and data capture for enumerators, field supervisors, research managers and lead scientists from the Makerere University Centre for Health and Population Research and a pilot testing of the data collection tool. Survey administration resulted in data collection for 8645 respondents aged 18 years and above. Specifically, 15.2% of the respondents had experienced chronic LBP in the last 12 months, of which 88.5% had experienced pain that was severe enough to affect their usual household, recreational or work activities. A total of 46% of this population in need of rehabilitation had been utilizing rehabilitation services (crude coverage), with no difference between women and men. Only 7.05% of the respondents with chronic primary low back pain experiencing limitations in functioning had been managed with sufficient quality to produce the desired health gain, defined by a minimal but meaningful improvement in functioning (effective coverage).

## 1. Introduction

Rehabilitation services are essential health services for optimizing functioning in individuals with health conditions in interaction with their environment [1]. Rehabilitation is therefore a critical health strategy to improve individual well-being, population health and societal welfare [2]. For service users, the expected impact of rehabilitation services is increased independence and societal participation. Many people, however, have limited access to the required rehabilitation services, particularly in low- and middle-income countries [3].

A recent study demonstrated that in the African region, approximately one in five people is living with a health condition that could benefit from rehabilitation. This need for rehabilitation significantly increased between 1990 and 2019, contributing to a 125.5% increase in years lived with a disability (YLDs). In Uganda specifically, in 2019, 6.8 million people experienced health conditions that are amenable to rehabilitation. This is largely due to musculoskeletal disorders, with an estimated 18,400 prevalent cases per 100,000 population [4]. Low back pain (LBP) accounts for 5% of all YLDs in Uganda. Studies show a high prevalence among specialized groups, such as 39.6% among health professionals in areas like Arua District [5]. Unfortunately, management often relies on medication and X-rays in clinics with limited resources [6], even though pain-relief techniques, pain counseling, and physical activity and exercise recommendations are often needed.

Rehabilitation is a public health priority for the government of Uganda. The existing workforce provides services across all National Referral Hospitals (NRHs) and Regional Referral Hospitals (RRHs) in both public and private sectors. Some rehabilitation services are also delivered in community settings through community outreach programs by partner non-governmental organizations. Rehabilitation services are free at the point of access in the public sector and have been considered in key national policy and planning documents within the Ministry of Health. These documents include the National Health Policy III (2021) and the latest Health Sector Development Plan 2021–2024. In addition, the National Rehabilitation and Assistive Technology Strategic Plan 2024–2029 is the first for Uganda and sets specific objectives for strengthening and mainstreaming rehabilitation, thereby guiding governmental and non-governmental stakeholders towards a common goal.

There is, however, no information available about the baseline health system performance of Uganda for rehabilitation—a gap that exists for most countries. For this, the measurement of effective (or quality-adjusted) coverage is recommended, preferably at the population level. Measuring effective coverage of rehabilitation means assessing whether a population that needs rehabilitation services receives interventions with sufficient quality to produce the desired health gain [7,8]. In contrast to crude coverage, which focuses solely on whether people use or have access to health interventions, effective coverage is a measure that combines intervention need, use, and quality. This is particularly useful for decision-makers because it measures a health outcome that can be linked to the healthcare budget.

This study aims to assess the effective coverage of rehabilitation in Uganda. We report on the first measurement of effective coverage in Uganda and globally using a World Health Organization (WHO) global tracer indicator [9]. The indicator uses a tracer health condition that was selected based on the following criteria: prevalence, disease burden in terms of functioning limitations, evidence base for rehabilitation interventions, and detectability through self-report questions. The indicator to measure effective coverage of rehabilitation at the population level is defined as the proportion of adults with chronic primary LBP experiencing limitations in functioning that benefit from rehabilitation. Chronic LBP is defined as pain and discomfort for more than 3 months located in the lumbar region or gluteal region with or without radiating leg pain. The pain is called primary or non-specific pain when no specific cause for the pain can be identified [10,11]. The quality of rehabilitation services is defined by the outcome: an improvement in functioning that is meaningful to service users, measured using the WHO Disability Assessment Schedule (WHODAS) 2.0 12-item tool, which utilizes a simple scoring system with a maximum score of 48 [9].

## 2. Materials and Methods

This section describes a study developed by the Makerere University Centre for Health and Population Research (MUCHAP) in Uganda and the WHO Rehabilitation Programme.

### 2.1. Study Design

A household survey was conducted incorporating the set of questions proposed by the WHO to assess effective coverage of rehabilitation services. The set of questions seeks to identify adults with chronic primary LBP, determine whether they had access to rehabilitation services, and evaluate whether these services have been successful. The survey questionnaire has three steps (Supplementary File S1).

In step 1, the questions seek to determine the denominator of the indicator by identifying people living with chronic primary LBP and experiencing limitations in functioning (component “rehabilitation need”). These questions ask about experiencing LBP in the last 12 months, the duration of the longest pain episode, whether the pain prevented the individual from carrying out his/her daily activities, and whether the individual had previously received surgery for the pain (to exclude people suffering from pain due to failed back surgery).

Respondents identified with chronic primary LBP proceed to steps 2 and 3 questions to determine rehabilitation service utilization and whether services have been of sufficient quality to produce the desired health gain (components “utilization” and “quality”). In step 2, respondents are asked a question to determine whether they had access to a health professional or a pain management program when experiencing continued LBP. For those who answer “yes” to this question, follow-up questions ask about whether these service providers aimed to help with better pain management, for example, when carrying out daily activities, or to restore function. The questions also capture whether the respondents have been receiving any of the following services: counseling about the pain and physical activities, pain-relief techniques, or psychological or exercise recommendations.

In step 3, respondents are asked to answer questions from the WHO Disability Assessment Schedule (WHODAS) 2.0 12-item. To answer these, respondents are requested to think about the difficulty they experienced (on average) while performing their daily activities in the last 30 days. The responses are categorized as none, mild, moderate, severe, extreme, or cannot do.

**Figure 1:**
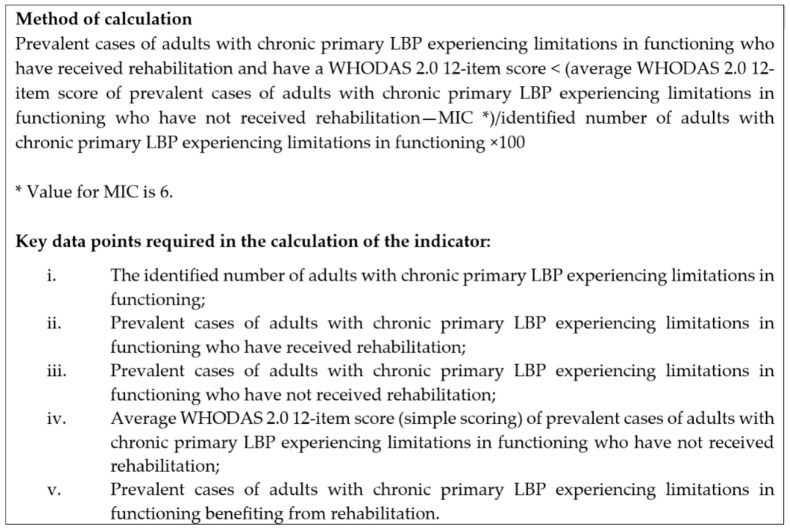
Calculation of the indicator for effective coverage of rehabilitation.

Following the data collection for the survey questions (Supplementary File S1), the indicator for effective coverage was calculated. The method of calculation and the key data points required in the calculation of the indicator are shown in Figure 1. Using the survey data, the indicator calculation is as follows: Respondents who answered “yes” to questions 1 and 3 and who answered “more than 3 months” to question 2 and “no” to question 4 define the number of people with chronic primary LBP experiencing limitations in functioning (data point i, denominator). The identified respondents answering “yes” to question 6 and “yes” to question 6a and/or 6b are then classified as individuals who have received rehabilitation (data point ii). To identify adults with chronic primary LBP and improved functioning following rehabilitation, the average WHODAS 2.0 12-item score of cases who did not receive rehabilitation (data point iii) is imputed as the baseline score for those receiving rehabilitation (data point iv). Individuals who have received rehabilitation and have a WHODAS 2.0 12-item score lower than this baseline WHODAS 2.0 12-item score minus 6 (minimal important change) are classified as having “benefited from services” (numerator, data point v).

### 2.2. Study Population, Location and Time

**Figure 2:**
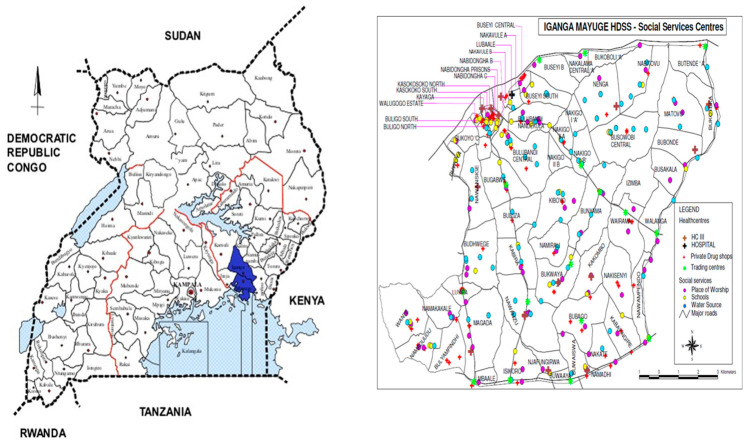
Map of Uganda and Iganga–Mayuge HDSS.

The set of questions was nested in the Iganga–Mayuge Health and Demographic Surveillance Site (IMHDSS) update round 22 that aimed to update population surveillance information on births, deaths and migration. The study questions were administered to individuals aged 18 years and above from households within the IMHDSS population cohort. The participants were drawn from the IMHDSS using its continuously updated, population-based household registry maintained through bi-annual censuses tracking births, deaths, migration, and key sociodemographic variables as the sampling frame, enabling structured random or stratified random selection rather than convenience sampling. The IMHDSS covers an area of 155 km^2^ made up of 65 villages in seven sub-counties in Eastern Uganda and distributed in Iganga and Mayuge districts (Figure 2). It is located about 120 km east of the capital city, Kampala, along the Uganda–Kenya highway. The population under surveillance of the IMHDSS is 94,568 people (2017 mid-year population), with 60% living in rural areas and the rest in peri-urban areas. It consists of 17,500 households, with an average household size of five individuals. The population has subsistence agriculture as the main occupation. The sex distribution is almost equal, with 51% females. The IMHDSS population is largely young, with more than 60% below 15 years of age [12].

Data collection by the enumerators commenced in June 2023 and ran for a period of four months, up to the end of September 2023.

### 2.3. Study Setup

The English version of the questions was translated into the local language, Lusoga, which is well understood by all residents in the HDSS. This was done by a field supervisor and lead scientist at MUCHAP, followed by a review from the research manager to ensure that the English meaning of the questions was maintained. The study questions were then developed in Open Data Kit (Kobo-collect) and embedded on an electronic tablet to aid data collection.

An online training session on survey administration and data capture was attended by the enumerators, field supervisors, research managers and lead scientists from MUCHAP. This training involved a review of the questions and study methodology. The enumerators could ask questions on areas that were not clear to them. The administration of the questions was then pre-tested in a neighboring village for consistency and suitability prior to actual data collection. Interviews were conducted in the Lusoga language. Data collection was jointly implemented by enumerators and their supervisors. Supervisors randomly assigned households, monitored adherence to training and standard procedures, observed interviews, and conducted spot checks to ensure data quality.

Collected data was submitted to the data server for synchronization on a daily basis. Study data was submitted as one complete dataset in MS Excel (csv). A data dictionary was attached to the dataset to guide the analysis. The dataset was anonymized, including only study identification numbers.

## 3. Results

Survey administration between June and September 2023 in the Iganga and Mayuge districts in rural Eastern Uganda resulted in data collection for 8645 respondents aged 18 years and above. The total eligible adult population within the IMHDSS is estimated at 45,000–50,000 persons, meaning that the recruited sample represents approximately 17–19% of all eligible adults. This is a substantial proportion that reflects the site’s known demographic profile (60% rural, 52% female, youthful age structure) and socioeconomic context without selection bias. We report no missing items. Respondents included 4855 females and 3790 males. Their age ranged from 18 to 110 years old. The age distribution of respondents was as follows: 1331 were between 18 and 30 years old; 4038 were between 31 and 50 years old; 2573 were between 51 and 70 years old; 654 were between 71 and 90 years old; and 49 were between 91 and 110 years old. About two-thirds (5695) were living in rural areas, and one-third (2950) were living in peri-urban areas. In terms of employment, 46% were in agriculture (farmers), 14% were unemployed, and 20% had a business (trade). Regarding education, 20% had never received an education, 46.5% had finished primary school, and 24.5% had finished secondary school. In terms of distribution for income levels based on wealth quintiles, 17.8% of respondents belonged to the lowest, 13.4% to the lower, 23% to the middle, 21% to the higher, and 22% to the highest wealth quintiles.

### 3.1. Identified Number of Adults with Chronic Primary LBP Experiencing Limitations in Functioning (Denominator)

A total of 44% (3797/8645) of the respondents had experienced pain (of any duration) in their lower back in the last 12 months. For these respondents, the distribution of the longest episode of pain in the lower back was relatively even: pain that lasted less than a month (n = 1028), between 1 and 3 months (n = 1454), and more than 3 months (n = 1315). People who experienced chronic LBP (for more than 3 months) were 70% females (n = 912) and 30% males (n = 403). In terms of age distribution, 5% were 18–30 years old, 10% were 31–50 years old, 22% were 51–70 years old, 38% were 71–90 years old, and 32% were 91–110 years old. Furthermore, 74% had never received an education or finished primary school, 58.5% were in agriculture, and 16.5% were unemployed. With respect to their level of income, people with chronic LBP were equally distributed across the different income level groups, from the lowest to the highest wealth quintile. Additionally, 88.5% had experienced pain that was severe enough to affect their usual household, recreational or work activities, and about 5% had received surgery for the pain. Therefore, we excluded 15% (n = 209) of people with chronic LBP from the denominator because they did not experience limitations in functioning and/or had received surgery for the pain.

In summary, 1106 of a total of 8645 respondents had experienced chronic LBP resulting in limitations in functioning in the last 12 months and had not received surgery. These 1106 respondents constitute the denominator of the indicator for effective coverage of rehabilitation (data point i).

### 3.2. Prevalent Cases of Adults with Chronic Primary LBP Experiencing Limitations in Functioning Benefiting from Rehabilitation (Numerator)

A total of 54.6% (604/1106) of identified respondents with chronic primary LBP experiencing limitations in functioning responded that they had consulted a health professional or accessed a pain management program when experiencing continued low back pain. Of these, 510 had received rehabilitation based on consulting service providers that aimed to help with better pain management or to recover function and/or received any of the listed rehabilitation services (data point ii). People receiving rehabilitation included 343 females and 167 males, with 341 living in rural areas and 169 living in peri-urban areas. A total of 250 were in agriculture, and 98 were unemployed; 345 had never received an education or had finished primary school; and 141 belonged to the lowest and lower wealth quintiles (Table 1).

Of the identified respondents accessing rehabilitation services (n = 510), 316 (62%) had consulted service providers who aimed to help with managing the pain and received at least one of the listed services. However, 173 (34%) had consulted service providers who aimed to help with managing the pain but did not receive any of the listed services, and 21 (4%) had received at least one of the listed services while thinking that the service providers were not aiming to help with better managing the pain.

**Figure 3:**
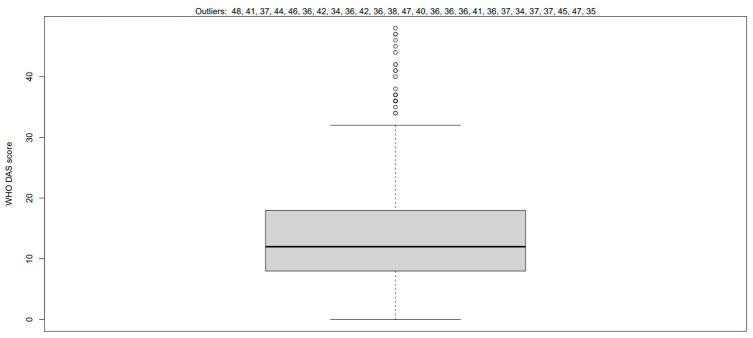
Distribution of WHODAS 2.0 12-item scores and interquartile range (IQR) for identified respondents who had not received rehabilitation.

A total of 53.8% (596/1106) of people with chronic primary LBP and limitations in functioning in the last 12 months had not received rehabilitation (data point iii). The average WHODAS 2.0 12-item score for these respondents was calculated at 13.92/48 (SD was 8.79) (data point iv). Since the data showed a skewed distribution of the WHODAS 2.0 12-item scores, the interquartile range (IQR), a measure of variability that describes the range between the first quartile and the third quartile, was estimated at 10 (Figure 3). Men who had not received rehabilitation had a mean WHODAS 2.0 12-item score of 13.21/48, and women had a mean score of 14.22/48, with no significant difference between the two. Furthermore, 4% (26/596) of WHODAS 2.0 12-item scores among respondents who had not received rehabilitation were considered outliers; these people lived in rural areas, 77% were women, and 73% did not consult a health professional. The mean WHODAS 2.0 12-item score excluding outliers was 12.76/48.

Of all identified respondents with chronic primary LBP and limitations in functioning that had received rehabilitation (n = 510), 78 (15%) had a WHODAS 2.0 12-item score that was lower than the average WHODAS 2.0 12-item score of individuals who had not received rehabilitation (13.92) minus the minimal important change for WHODAS 2.0 12-items (value is 6) (numerator, data point v). These 78 respondents constitute the numerator of the effective coverage indicator. They had an average WHODAS 2.0 12-item score of 4.7/48. Of these, 51 were females, and 27 were males; 54 lived in rural areas, and 24 lived in peri-urban areas; 37 were in agriculture, and 10 were unemployed; 52 had never received education or had finished primary school, and 20 belonged to the lowest and lower wealth quintiles (Table 2).

## 4. Discussion

In this study, for the first time, the WHO global tracer indicator for effective coverage of rehabilitation was measured at the population level using chronic primary LBP as the tracer health condition. The survey administration was conducted in rural Eastern Uganda in 2023 and estimated a 12-month prevalence of chronic LBP of 15.2%, with 70% females and 30% males. In total, 1106 of 8645 respondents represent the population in need of rehabilitation interventions for the indicator (denominator). Based on the definition of accessing rehabilitation, 46% had been utilizing rehabilitation services (crude coverage). There was no difference in the crude coverage of rehabilitation services between women and men (*p* = 0.5699). For people living in rural or peri-urban areas, however, accessibility to rehabilitation was different, with 40.5% in rural and 64% in peri-urban areas (*p* < 0.001). Rehabilitation services coverage was significantly higher for people who were unemployed (50.7%) than for those who were in agriculture (30%), for people who had finished secondary school or higher education (58%) compared to people who had never received an education (42.4%), and for people from the highest wealth quintile (69%) compared to those from the lowest wealth quintile (33.3%).

With regard to effective coverage, 78 of 1106 identified respondents in need of rehabilitation were managed with sufficient quality to produce a desired health gain based on an improvement in functioning that is meaningful to service users (numerator). Effective coverage of rehabilitation, therefore, was 7.05%. For women, this was 6.7%, and for men, 7.8% (*p* = 0.5699). In rural and peri-urban areas, 6.4% and 9% of identified respondents, respectively, were effectively managed with rehabilitation services (*p* = 0.1787). Effective coverage of rehabilitation services was 5.2% for people who were unemployed and 4.4% for those who were in agriculture, compared to 40.2% for people with other occupations (*p* < 0.05). Additionally, 9.1% of people who had finished secondary school or higher education were effectively managed compared to 5.7% of people who had never received an education (*p* = 0.2601). Effective coverage was 11.7% for people from the highest wealth quintile compared to 3.6% for people from the lowest wealth quintile (*p* < 0.05).

Rehabilitation is an integral part of UHC, and low back pain is a leading cause of years lived with disability globally and a major economic burden for many health systems and societies [13]. Therefore, as effective rehabilitation interventions are available to manage chronic primary LBP, it would be meaningful to make them accessible through the Ugandan health system as part of UHC [9]. Uganda is committed to achieving UHC by 2030 and has made improvements in the UHC effective coverage index of 3.1% annually. The commitment to UHC is enshrined in recent national policies, in which the government sets out its aim to implement a universal health insurance system, improve the quality of care, and reduce medical referrals abroad to increase coverage [14]. In Uganda, many people with chronic primary LBP could benefit from quality rehabilitation services. The estimated prevalence of chronic LBP of 15.2% is comparable to estimates from other countries with different income levels [11,15,16]. Interventions for the rehabilitation of the condition have been recommended in WHO normative products [17]. In particular, the rehabilitation management of chronic primary LBP at the primary healthcare level is the subject of a recent WHO guideline [18]. The management of acute LBP patients based on a risk-group classification at the primary healthcare level has also been proven to be successful in terms of reducing pain intensity, healthcare use, and time off work [19,20,21]. Whether this approach in managing acute LBP patients is effective in preventing patients from transitioning to chronic LBP remains to be studied.

Crude coverage of rehabilitation for people with chronic primary LBP in rural Uganda corresponds with recent estimates of unmet rehabilitation needs in LMICs, where up to 50% of people are not receiving the rehabilitation they need [3]. In LMICs, the main reasons for unmet needs for rehabilitation are the absence or unequal geographical distribution of services, lack of transportation, and the unaffordability of services [2,22]. A lack of referral, including a lack of understanding and information sharing about the benefits of rehabilitation [23], has been identified as a common barrier to rehabilitation service utilization in both LMICs and HICs [24]. Strong governance, financial commitment, and evidence-based planning for integrating rehabilitation into primary healthcare, however, have been shown to be effective in increasing access to rehabilitation [25]. With about five rehabilitation professionals employed in the Iganga and Mayuge districts (0.52 per 10,000 population), this study’s crude coverage estimation of 46% was much higher than expected. Certainly, this is partially due to the method used to measure access to rehabilitation services in the population survey; it does not ask about access to specific rehabilitation professionals. This means that at least some respondents were accessing rehabilitation services through task-sharing approaches. This echoes the work by Odokonyero Tonny et al., which shows fundamental challenges with the health infrastructure needed to deliver UHC in Uganda. Spatial inequality in health facility population coverage and low health workforce densities imply that the existing health workforce is deficient, and alternate models of care are needed to expand population-based healthcare services [26]. Apart from the poor availability of rehabilitation services, the interacting effects of factors like socioeconomic status, racial or ethnic identity, lack of referral, and inadequate insurance on rehabilitation service utilization in Uganda have to be studied.

A total of 7.05% of people identified as having a need for rehabilitation services in 2023 received interventions with sufficient quality to produce the desired health gain, with no significant difference between men and women. This is in contrast to findings in HICs, where rehabilitation services use, adherence, and outcomes have been reported as worse for women than men [27]. In any case, there was a significant drop from crude coverage (46%) to quality-adjusted coverage (7.05%) in Uganda. This may indicate that implementation of health services does not necessarily equate to a health gain for the population, although some evidence exists for achieving better health outcomes with improved healthcare coverage [26]. In this context, it is important to note that rehabilitation services, in contrast to, e.g., immunization services, often involve complex interventions, with outcomes very much depending on available infrastructure, trained professionals and coordination of care. When comparing our estimate of effective coverage with the effective coverage of maternal and child health services across eight high-mortality countries in Africa, including Uganda, effective coverage of rehabilitation is lower than the effective coverage for antenatal care, family planning and sick-child care [28]. Specifically, in the Oyam district, Uganda, effective coverage is 40% for antenatal care visits, 48% for institutional deliveries, and 77% for postnatal care visits [29]. Regarding the latter, it is, however, important to note that facility audits have been utilized for determining the quality of health services delivered, as opposed to measuring the outcome of healthcare provided (rehabilitation estimate). Cross-sectional mixed-methods studies, utilizing both qualitative and quantitative approaches, are needed to identify barriers to effective provision of rehabilitation services in rural Uganda, including patient characteristics such as occupation, socioeconomic status, or underlying health conditions [30]. Compliance with standards of rehabilitation practice has been modest in LMICs, in general, and suggests that there is a need for an emphasis on the quality performance of rehabilitation services [31].

The imputed baseline functioning score for respondents with chronic primary LBP in this study corresponds to pre-rehabilitation scores that have been described in HICs [32]. The indicator’s method of measurement uses the average WHODAS 2.0 12-item score (simple scoring) of cases in the population cohort who did not receive rehabilitation as the baseline functioning score for those who have utilized rehabilitation services. This baseline score was calculated at 13.92/48 (SD = 8.79). A study by Wong et al. to identify WHODAS 2.0 12-item scores for people with chronic LBP seeking and accessing rehabilitation reported mean pre-rehabilitation scores of 11.4/48 (SD = 8.7) or 14.4/48 (SD = 9.4) using simple scoring and 25.8/100 (SD = 2.2) using complex scoring [32]. These data from HICs correspond to the calculated pre-rehabilitation score of cases who received rehabilitation in the population cohort of rural Uganda. Measurement with the WHODAS 2.0 12-item showed that individuals with chronic LBP seeking rehabilitation have moderate limitations in functioning.

The effective coverage of rehabilitation for people with chronic primary LBP is a good tracer measure of a health system’s performance for rehabilitation but, obviously, cannot be used to represent the whole rehabilitation sector. There is a wide range of health conditions amenable to rehabilitation, and therefore a long list of rehabilitation interventions exists to manage those conditions [17]. Looking at the range of health condition groups in need of rehabilitation, these include musculoskeletal, neurological, respiratory, cardiovascular, and mental health conditions; hearing impairment; vision impairment; and cancer. For each of these health conditions, a specific model of rehabilitation care with its own quality features should be applied. This may include the provision of services at higher levels of healthcare for people with acute and complex rehabilitation needs (such as for people with a new-onset stroke, amputation, or spinal cord injury, among others); the development of service delivery models closer to people’s homes for chronic conditions; and dedicated rehabilitation centers providing highly specialized inpatient and outpatient services for children suffering from severe disabilities [33,34]. This important diversity in rehabilitation services delivery requires the strengthening of all levels of healthcare [35]. While the measurement of effective coverage of rehabilitation for primary chronic LBP may be used as a proxy for the assessment of a health system’s performance for rehabilitation, more rehabilitation interventions and health conditions that require rehabilitation should be included in the measurement of effective coverage. This is, however, often not possible at the population level because most health conditions do not fulfill the criteria used for tracer health condition selection. In response, other WHO work on health facility reporting has recently recommended indicators for assessing the coverage of rehabilitation for people experiencing a sudden onset of functional decline resulting from acute conditions that require intensive inpatient rehabilitation services [36].

## 5. Limitations

A limitation of this study relates to the translation process for the survey questions into the local language, which did not include back-translation. A research manager at MUCHAP, however, independently verified the translated questions to ensure that the meaning was maintained. Enumerators and field supervisors also received training in order to ensure their comprehension of the questions and response options.

We acknowledge that, as a sub-study nested within a defined surveillance area, generalization beyond populations with similar demographic profiles should be made with appropriate caution.

The validity of the approach used to estimate effective coverage, particularly the definition of individuals who benefited from rehabilitation, should be interpreted with caution. This is a cross-sectional study using a cutoff for the “benefit of rehabilitation”. This approach may be in contrast with clinical expertise, where individual patients may not experience such a high level of functioning following rehabilitation yet benefit from services because they experienced severe limitations in functioning at baseline. When calculating an estimate of effectiveness at the population level, this is, however, accepted based on the aggregation of data using a conservative approach for a larger sample in the population. In addition, an important caveat when comparing health outcomes of individuals with and without exposure to the interventions is the presence of unmeasured confounding, as the analyses are observational in nature, and confounding needs to be accounted for. Potential confounding factors—such as rehabilitation dosage and psychosocial risk factors—may affect both rehabilitation utilization and functional outcomes. A recall bias may also be present, as the analysis relies on self-reported data.

## 6. Conclusions

This paper presents, for the first time, a measurement for the WHO global tracer indicator for effective coverage of rehabilitation. At the population level, it measures the proportion of adults with chronic primary LBP experiencing limitations in functioning that benefit from rehabilitation. In rural Uganda, in 2023, this effective coverage measurement for rehabilitation was estimated at 7.05%.

## Figures and Tables

**Table 1:** Coverage of rehabilitation services for people with chronic primary LBP (46%, n = 510/1106) by stratifier.

Age (*p* = 0.0067)	18–30 Years	31–50 Years	51–70 Years	71–90 Years	91–110 Years
	49% (n = 26/53)	37.5% (n = 124/330)	49.5% (n = 243/491)	51% (n = 111/218)	43% (n = 6/14)
**Sex** **(*p* = 0.3049)**	Female	Male			
	45% (n = 343/762)	48.5% (n = 167/344)			
**Residence** **(*p* < 0.001)**	Rural	Peri-urban			
	40.5% (n = 341/842)	64% (n = 169/264)			
**Occupation** **(*p* < 0.001)**	Agriculture	Unemployed	Other (trade, labor, transport)		
	30% (n = 250/836)	50.7% (n = 98/193)	48% (n = 37/77)		
**Education** **(*p* < 0.001)**	Never received	Primary school	Secondary school and above		
	42.4% (n = 117/276)	41.8% (n = 228/545)	58% (n = 165/285)		
**Wealth quintile** **(*p* < 0.001)**	Lowest	Lower	Middle	Higher	Highest
	33.3% (n = 74/222)	33.8% (n = 67/198)	44.5% (n = 124/279)	53.5% (n = 115/215)	69% (n = 118/171)

**Table 2:** Effective coverage of rehabilitation services for people with chronic primary LBP (7.05%, n = 78/1106) by stratifier.

Age (*p* = 0.0541)	18–30 Years	31–50 Years	51–70 Years	71–90 Years	91–110 Years
	9.5% (n = 5/53)	8.5% (n = 28/330)	8% (n = 39/491)	2.7% (n = 6/218)	0% (n = 0/14)
**Sex** **(*p* = 0.5699)**	Female	Male			
	6.7% (n = 51/762)	7.8% (n = 27/344)			
**Residence** **(*p* = 0.1787)**	Rural	Peri-urban			
	6.4% (n = 54/842)	9% (n = 24/264)			
**Occupation** **(*p* < 0.00001)**	Agriculture	Unemployed	Other (trade, labor, transport)		
	4.4% % (n = 37/836)	5.2% (n = 10/193)	40.2% (n = 31/77)		
**Education** **(*p* = 0.2601)**	Never received	Primary school	Secondary school and above		
	5.7% (n = 16/276)	6.6% (n = 36/545)	9.1% (n = 26/285)		
**Wealth quintile** **(*p* = 0.0346)**	Lowest	Lower	Middle	Higher	Highest
	3.6% (n = 8/222)	6% (n = 12/198)	6.5% (n = 18/279)	7% (n = 15/215)	11.7% (n = 20/171)

## Data Availability

The raw data supporting the conclusions of this article will be made available by the authors on request.

## Supplementary Materials

The following supporting information can be downloaded at https://www.mdpi.com/article/10.3390/ijerph23060693/s1, Supplementary file S1. Questions to provide key data points for calculating effective coverage of rehabilitation for chronic primary LBP.

## Abbreviations

The following abbreviations are used in this manuscript:

LBP	Low back pain
LMIC	Low- and middle-income countries
HIC	High-income countries
IMHDSS	Iganga–Mayuge Health and Demographic Surveillance Site
MUCHAP	Makerere University Centre for Health and Population Research
UHC	Universal health coverage
WHO	World Health Organization
WHODAS	World Health Organization Disability Assessment Schedule
