# Supplementary material for: Effective Coverage of Rehabilitation for Adults with Chronic Primary Low Back Pain in Uganda
Source: Int J Environ Res Public Health. 2026 May 23;23(6):693. doi: 10.3390/ijerph23060693 (PMC13299537; doi:10.3390/ijerph23060693)
Supplement: Supplementary file 1 [file ijerph-23-00693-s001.zip › ijerph-4232244-supplementary.pdf]

Supplementary file S1. Questions to provide key data points for calculating effective coverage of rehabilitation for chronic primary LBP.

Step 1: Ask all participants the following questions:

1. During the last 12 months, was there a time that you had pain in your lower back (in the area shown in the figure*)? Yes/No (if No, leave the questionnaire)
2. How long did the longest episode of this pain in your lower back last, considering that you may have experienced several episodes of pain and that pain intensity may have varied?
-Less than a month/between 1 and 3 months/more than 3 months
3. During this episode of pain in your lower back, has the pain been severe enough to affect your usual household, recreational or work activities? Yes/No
4. At any point in time, did you receive an operation for this pain in your lower back? Yes/No

Step 2: **Ask the following questions to all respondents who have answered 'Yes' to questions 1 and 3, and who answered 'more than 3 months' to question 2 and 'No' to question 4:**

5. Regarding this episode of low back pain, did you receive any pain medication at the onset? -Yes/No
6. In addition, when experiencing continued low back pain, did you consult a health professional or access a pain management program? Yes/No

If Yes,

6a. Did these service providers aim to help you better care for your pain, for example, when carrying out daily activities, or to recover function? Yes/No
6b. Have you received any of the following services: counseling about your pain and physical activities, pain relief techniques, or psychological or exercise recommendations? Yes/No

Step 3: **Administer WHODAS 2.0 12-item to all respondents who have answered Step 2 questions:**

In the past 30 days, how much difficulty did you have in:	None	Mild	Moderate	Severe	Extreme or cannot do
Standing for long periods such as 30 minutes?	0	1	2	3	4
Taking care of your household responsibilities?	0	1	2	3	4
Learning a new task, for example, learning how to get to a new place?	0	1	2	3	4

How much of a problem did you have joining in community activities (for example, festivities, religious or other activities) in the same way as anyone else can?	0	1	2	3	4
How much have you been emotionally affected by your health problems?	0	1	2	3	4
Concentrating on doing something for ten minutes?	0	1	2	3	4
Walking a long distance such as a kilometer [or equivalent]?	0	1	2	3	4
Washing your whole body?	0	1	2	3	4
Getting dressed?	0	1	2	3	4
Dealing with people you do not know?	0	1	2	3	4
Maintaining a friendship?	0	1	2	3	4
Your day-to-day work/school?	0	1	2	3	4

* Figure from the Global Alliance for Musculoskeletal Health Survey, with permission (question 1).

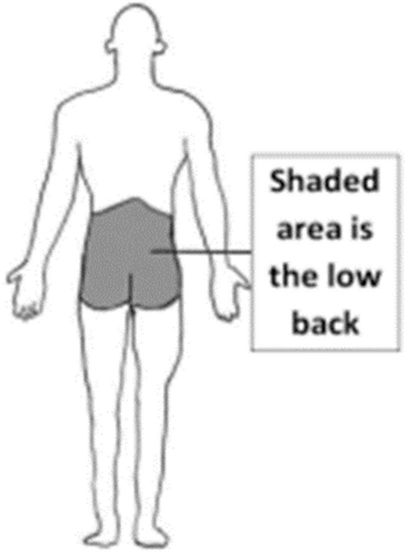
Shaded area is the low back
